# Supplementary material for: Reconstructing lost BOLD signal in individual participants using deep machine learning
Source: Nat Commun. 2020 Oct 7;11:5046. doi: 10.1038/s41467-020-18823-9 (PMC7542429; doi:10.1038/s41467-020-18823-9)
Supplement: Supplementary file 1 — Supplementary Information [file 41467_2020_18823_MOESM1_ESM.pdf]

Supplementary Information

Reconstructing lost BOLD signal in individual participants using deep machine learning

Yuxiang Yan[1,2†], Louisa Dahmani[1,3†], Jianxun Ren[1,4†], Lunhao Shen[1,4], Xiaolong Peng[1], Ruiqi Wang[1], Changgeng He[1,4], Changqing Jiang[4], Chen Gong[4], Ye Tian[4], Jianguo Zhang[5], Yi Guo[6], Yuanxiang Lin[7], Shijun Li[1], Meiyun Wang[3]*, Luming Li[4,8]*, Bo Hong[2]*, Hesheng Liu[1,8,9]*

[1]Athinoula A. Martinos Center for Biomedical Imaging, Department of Radiology, Massachusetts General Hospital, Harvard Medical School, Charlestown, MA, USA.
[2]Department of Biomedical Engineering, School of Medicine, Tsinghua University, Beijing, China.
[3]Department of Radiology, Zhengzhou University People Hospital & Henan Provincial People's Hospital, Zhengzhou, China.
[4]National Engineering Laboratory for Neuromodulation, School of Aerospace Engineering, Tsinghua University, Beijing, China.
[5]Department of Neurosurgery, Tiantan Hospital, Capital Medical University, Beijing, China.
[6]Department of Neurosurgery, Peking Union Medical College Hospital, Beijing, China.
[7]Department of Neurosurgery, First Affiliated Hospital of Fujian Medical University, Fuzhou, China.
[8]Beijing Institute for Brain Disorders, Capital Medical University, Beijing, China.
[9]Department of Neuroscience, Medical University of South Carolina, Charleston, SC, USA.

† These authors contributed equally
*These authors jointly supervised this work

To whom correspondence should be addressed. E-mail: hesheng.liu@mgh.harvard.edu or hongbo@tsinghua.edu.cn

Supplementary Figures

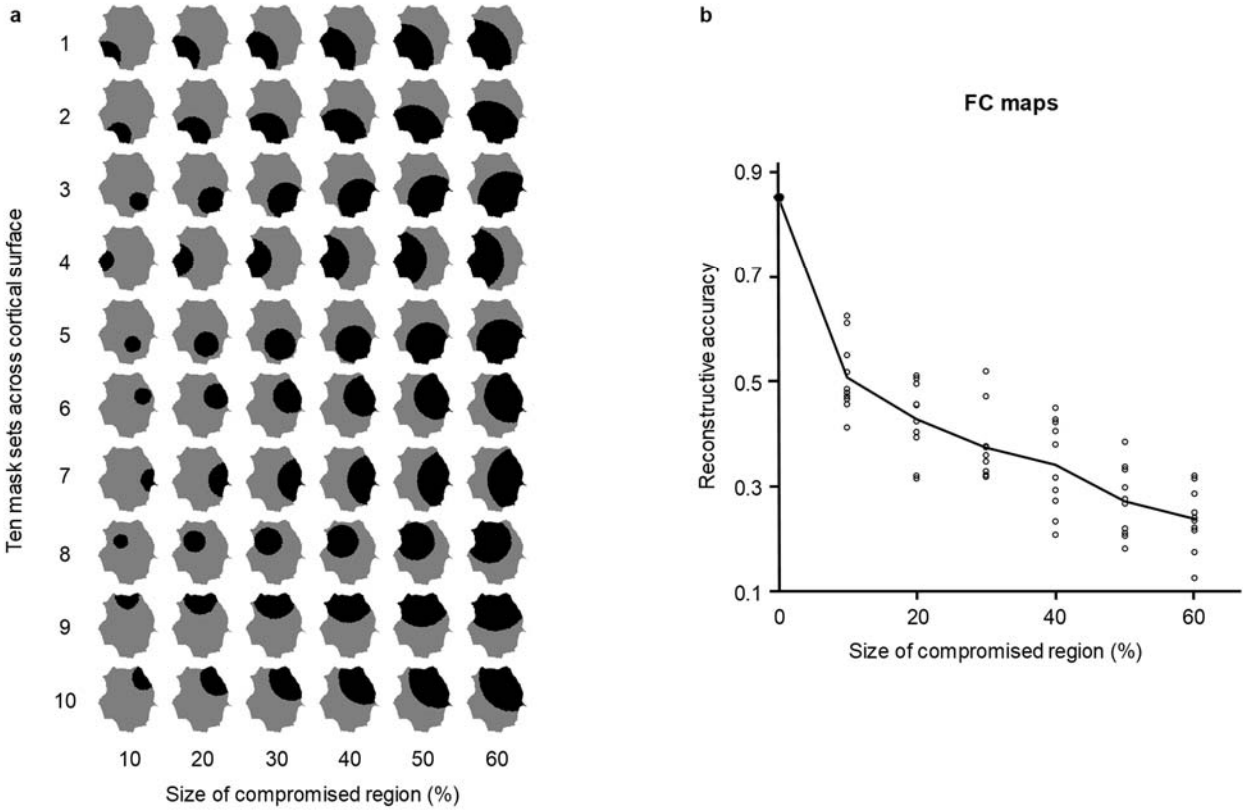

Supplementary Fig. 1 Reconstructive accuracy varies according to the size of the compromised area. a Ten vertices on the cortical surface were selected at random and served as the center of their respective compromised regions (indicated in black). From that center, we removed BOLD signals in incrementally larger regions, which spanned 10-60% of the cortical surface (with steps of 10%). The vertices nearest the centers were included in each of the masks. **b** This graph shows reconstructive accuracy as a function of the size of the compromised regions, as measured by calculating the average correlation between the original and reconstructed functional connectivity (FC) maps within that region. When we feed the DCGAN model an intact frame (size = 0%), the reconstructive accuracy is $r = 0.85 \pm 0.00$. When the compromised region spans 10% of the cortical surface, the accuracy is $r = 0.51 \pm 0.07$. From there, each incremental increase of 10% reduces the accuracy in a linear fashion ($F(2.62,23.55) = 93.68$, $p < 0.001$). Data points indicate the average reconstructive accuracy for each of the 10 cortical masks. Source data are provided as a Source Data file.

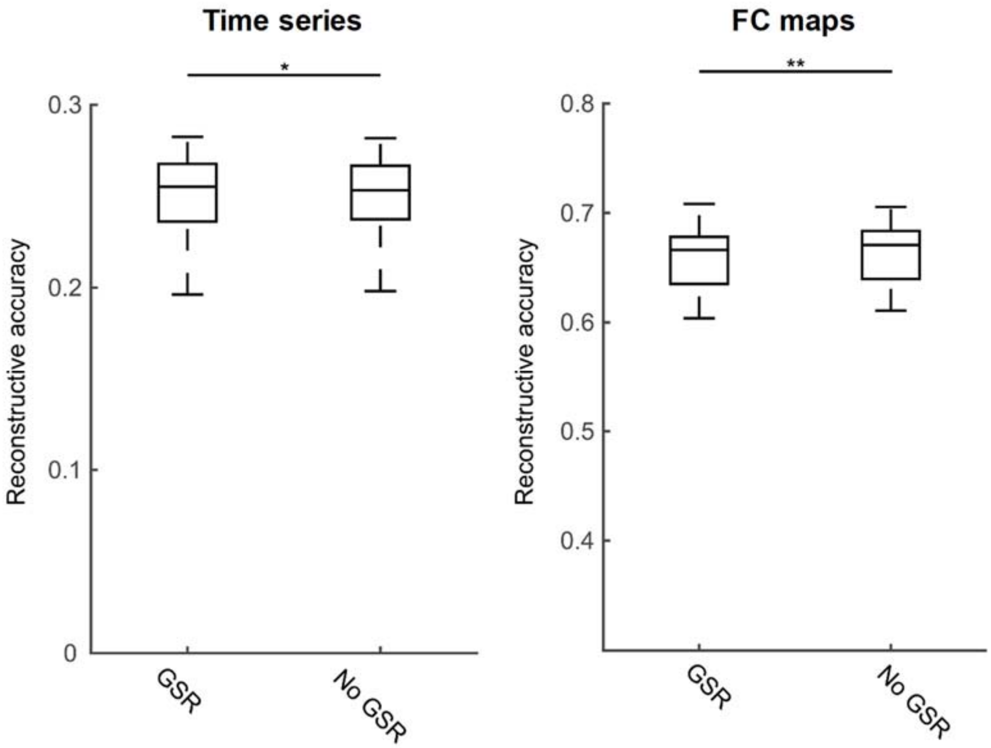

Supplementary Fig. 2 Reconstructive accuracy is stable regardless of whether global signal regression is applied to the data. Box-and-whisker plots are shown, with the center line indicating the median, box limits indicating upper and lower quartiles, and whiskers indicating 1.5 times the interquartile range. Compared to data preprocessed with global signal regression (GSR; n = 20), data preprocessed without GSR (n = 20) yielded significantly lower reconstructive accuracy for time series (GSR mean = 0.27; No GSR mean = 0.27; $t(19) = -2.81$, $p = 0.01$) and significantly higher reconstructive accuracy for FC maps (GSR mean = 0.66; No GSR mean = 0.66; $t(19) = 4.87$, $p < 0.001$). Crucially, the effect is so small (mean differences in *r* coefficients: -0.001 for time series and 0.003 for FC maps) that it is deemed inconsequential. GSR therefore has no meaningful impact on DCGAN reconstructive accuracy. All statistical tests were two-sided and corrected for multiple comparisons. Source data are provided as a Source Data file.

*: $p \leq 0.05$

**: $p \leq 0.001$

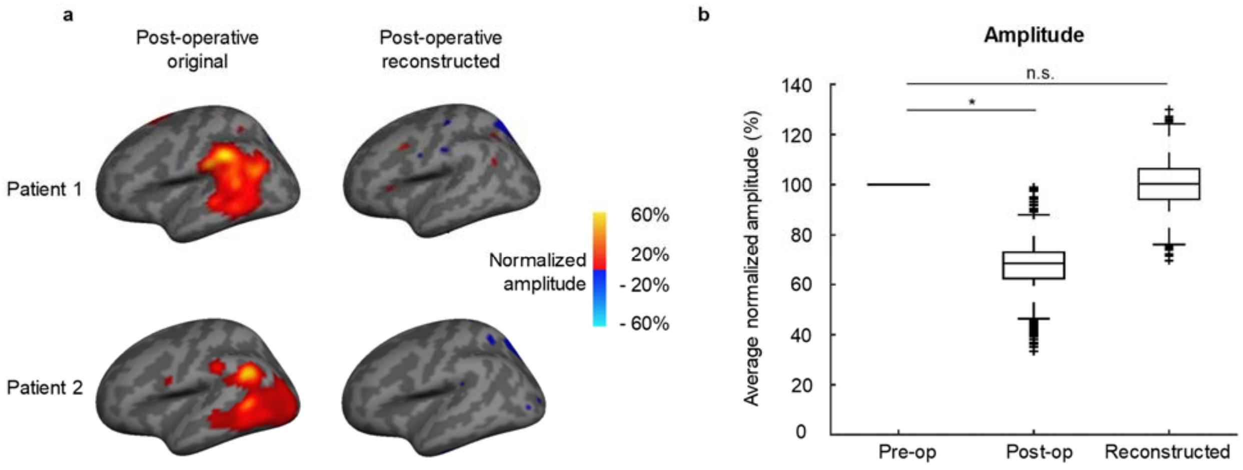

Supplementary Fig. 3 The BOLD signal amplitude is restored in DCGAN-reconstructed frames. a We investigated whether there was any residual loss of BOLD signal in the DBS-compromised regions in patients with Parkinson's disease after reconstruction. The left column shows the signal amplitude loss in the compromised region of two representative patients, calculated by subtracting the post-operative BOLD signal from the pre-operative BOLD signal. Both patients exhibit substantial signal loss following implantation. In contrast, the reconstructed BOLD signal shows no residual loss (right column). **b** The graph shows the normalized BOLD amplitudes within the compromised regions across all patients in the clinical sample (N = 12). Box-and-whisker plots are shown, with the center line indicating the median, box limits indicating upper and lower quartiles, whiskers indicating 1.5 times the interquartile range, and plus signs indicating outliers. The post-operative BOLD amplitudes are substantially and significantly lower than the pre-operative BOLD amplitudes ($t(5138)=-165.78$, $p<0.001$). The reconstructed BOLD amplitudes are not significantly different from the pre-operative BOLD amplitudes ($t(5138)=1.647$, $p=0.10$). The average signal amplitude increased from 67.44±8.53 post-operatively to 100.32±9.04) after reconstruction. All statistical tests were two-sided and corrected for multiple comparisons. Source data are provided as a Source Data file.
*: $p \leq 0.001$
n.s.: not significant

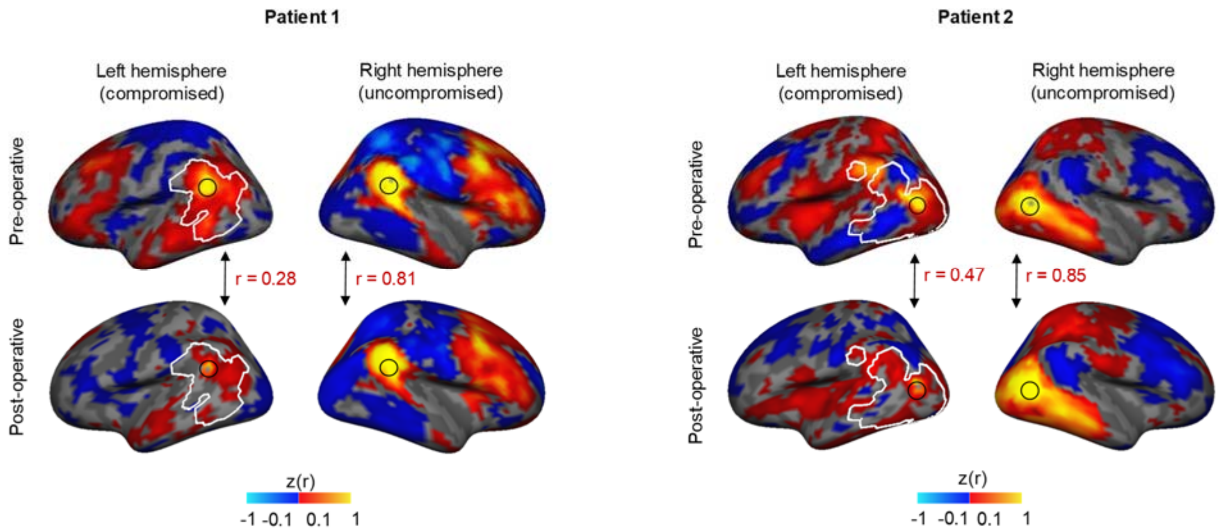

Supplementary Fig. 4 FC maps remain stable after electrode implantation. Here we show proof of concept that FC maps remain stable following electrode implantation surgery. We generated pre- and post-operative FC maps from one seed (black circle) in the compromised region (left hemisphere, outlined in white), and the same seed in the uncompromised region in the right hemisphere. FC maps are shown here for two representative patients. The left hemisphere post-operative FC map presents substantial differences from the pre-operative FC map, as indicated by a low correlation of $r = 0.28$ in Patient 1 and $r = 0.47$ in Patient 2. When looking at the uncompromised seed in the right hemisphere, the post-operative FC map is highly similar to the intact pre-operative FC map, as shown by a correlation of $r = 0.81$ in Patient 1 and $r = 0.85$ in Patient 2. Thus, functional connectivity does not seem substantially affected by electrode implantation.
